# Emerging lessons from the COVID-19 pandemic about the decisive competencies needed for the public health workforce: A qualitative study

**DOI:** 10.3389/fpubh.2022.990353

**Published:** 2022-09-02

**Authors:** Osnat Bashkin, Robert Otok, Lore Leighton, Kasia Czabanowska, Paul Barach, Nadav Davidovitch, Keren Dopelt, Mariusz Duplaga, Leah Okenwa Emegwa, Fiona MacLeod, Yehuda Neumark, Maya Peled Raz, Theodore Tulchinsky, Zohar Mor

**Affiliations:** ^1^Department of Public Health, Ashkelon Academic College, Ashkelon, Israel; ^2^The Association of Schools of Public Health in the European Region (ASPHER), Brussels, Belgium; ^3^Department of International Health, Care and Public Health Research Institute CAPHRI, Faculty of Health, Medicine & Life Sciences, Maastricht University, Maastricht, Netherlands; ^4^College of Population Health, Thomas Jefferson University, Philadelphia, PA, United States; ^5^College of Population Health, Sigmund Freud University, Vienna, Austria; ^6^Faculty of Health Sciences, Department of Health Policy and Management, School of Public Health, Ben Gurion University of the Negev, Beersheba, Israel; ^7^The Israeli Association of Public Health Physicians (IPAPH), Israeli Medical Association, Ramat Gan, Israel; ^8^Faculty of Health Sciences, Department of Health Promotion and e-Health, Institute of Public Health, Jagiellonian University Medical College, Krakow, Poland; ^9^Department of Health Sciences, The Swedish Red Cross University College (SRCUC), Huddinge, Sweden; ^10^School of Public Health, University College Cork, Cork, Ireland; ^11^Braun School of Public Health and Community Medicine, Hebrew University-Hadassah, Jerusalem, Israel; ^12^Faculty of Social Welfare and Health Sciences, School of Public Health, University of Haifa, Haifa, Israel

**Keywords:** public health workforce, leadership, competencies, management, communication, health promotion

## Abstract

The global COVID-19 crisis exposed the critical need for a highly qualified public health workforce. This qualitative research aimed to examine public health workforce competencies needed to face COVID-19 challenges and identify the gaps between training programs and the competency demands of real-world disasters and pandemics. Through a sample of thirty-one participant qualitative interviews, we examined the perspectives of diverse stakeholders from lead public health organizations in Israel. Grounded Theory was used to analyze the data. Six themes emerged from the content analysis: public health workforce's low professional status and the uncertain future of the public health workforce; links between the community and Higher Education institutions; the centrality of communication competencies; need to improve health promotion; the role of leadership, management, and partnership, and innovation in public health coherence. Increasing the attractiveness of the profession, professional and financial support, and improving the working conditions to ensure a sustainable and resilient PH system were deemed necessary. This paper describes and cultivates new knowledge and leadership skills among public health professionals, and lays the groundwork for future public health leadership preparedness programs.

## Introduction

The COVID-19 pandemic crisis poses unique and unchartered challenges to public health (PH) systems worldwide (1). PH professionals needed to respond swiftly while making organizational adjustments to manage the pandemic, including disease monitoring and data analysis, health education and promotion, epidemiological investigations, setting guidelines, and communicating challenging messages to engage and build public trust. PH professionals faced an unprecedented workload, bodily and emotional abuse, and escalating dangers, emphasizing the critical needs of a qualified workforce (2).

Ensuring effective responses to large-scale outbreaks requires nimbly adaptive health systems capacities and strengthening public health workforce (PHW) competencies to meet population health needs (3). Analyzing the emergent PH needs and aligning training programs to fill workforce gaps are essential in the post-COVID era. Analysis of job postings before and during the pandemic found that the demand for PH competencies in the USA has soared (4). The authors demonstrate that the most significant increase in job demands during the pandemic was for roles requiring competencies in applied research and epidemiology, technology management, and community health.

A recent study in Korea found that PH nurses scored low in competencies clustered around the three disaster management stages: prevention stage, mitigation stage, and recovery post-disaster stage, using the Disaster Preparedness Evaluation Tool (5). Disaster management competencies exhibited a positive correlation with age and job satisfaction and a negative correlation with depression, stress, and burnout among PH nurses. This reflects the need to develop professional disaster competencies and urgently improve the work environment. Czabanowska and Kuhlman (6) call for a change in the traditional PH core competencies and an expansion of PH workers (PHW) scope to focus on strengthening PHW leadership and competencies related to emergency preparedness, such as digital competencies (7), innovative epidemiological competencies (4), and emergency risk and crisis communication competencies (8). Ghaffar et al. (9) highlight the need to strengthen community engagement and trust-building and underscore the need to develop new areas of knowledge related to pandemic responses, harmful effects associated with the spread of misinformation, and innovative data collection technologies.

The COVID-19 crisis called attention to the significance of specific PH competencies in addressing the needs of vulnerable at-risk populations such as minorities, seniors and adapting recommendations and messaging to the local contexts of communities at risk (10). Psychosocial competencies need to be strengthened, including supporting a deeper understanding and empathy in light of COVID-19 imposed restrictions, sociologic challenges of homelessness, food insecurity, and limited access to healthcare for vulnerable populations (11). We conducted in-depth interviews with key PH stakeholders and policymakers to gain deeper insights into the PH workforce competencies and employers' needs in Israel.

## Materials and methods

### Setting and participants

We conducted a prospective, qualitative study as part of a multinational Erasmus Plus Capacity Building European Union-funded grant in Higher Education funded project entitled “Sharing European Educational Experience in Public Health for Israel (SEEEPHI): harmonization, employability, leadership, and outreach (12).

#### The Israeli health system

Israel's health system is predominantly a public system designed to care for almost 10 million citizens (13). All residents are entitled to comprehensive health insurance, with self-selected membership in one of four non-for-profit Health Funds that provide a nationally defined “basket of services” under the supervision of the Ministry of Health (MoH) (14). The MoH is responsible for the regulation of the health system and facilitates PH services. The structure of the PH system includes the headquarters units responsible for policymaking and issuing guidelines to regional health departments that provide community-based services and operate mother and child health clinics. The regional departments are operated by physicians with PH expertise, public health nurses, environmental epidemiologists, and other public health-related professionals.

#### Participants

Thirty-one mid to senior-level managers in the Israeli PH service, health funds, and hospitals were interviewed between September and December 2021. We used purposive sampling to ensure a diversity of respondents and selected to obtain maximal subjective heterogeneity and serve as rich information sources to meet the study's objectives. The sample included interviewees employed in PH and health services, health funds, and hospitals and represented all the health districts in Israel (Supplementary Datasheet 1).

### Data collection

We conducted a prospective qualitative study using a pre-tested semi-structured individual interviews using interview guides. The topics that guided the question development are the following: (1) PHW competencies needed to address COVID-19 challenges and prepare the PH system for the post-pandemic period and future health challenges; and (2) potential gaps between academic training and the demands of the PH labor market in Israel.

The interview guide was based on the WHO-ASPHER Competency Framework for the Public Health Workforce in the European Region (CFPHW tool) (15). This validated tool defines 10 competency sections focusing on three major categories: content and contexts, relations and interactions, performance, and achievements. The framework enables standardization and consistent definition of the competencies required by PH professionals. The interview guide was validated using the content validation method, by three PH experts (one from PH services and two from PH higher education programs), to ensure that the questions were relevant to PH workforce competencies and the needs of the Israeli PH system needs. The guide was pilot tested with two senior PH managers to ensure a smooth interview flow and verify comprehension of the questions. Information collected during the interviews included deficiencies in the current Israeli PH training programs, the influence of COVID-19 on the PHW, the needs of the health system and the community, requirements for improved field competencies, and background demographic details of the interviewee. Interviewees were invited to share their recommendations regarding how they would improve and expand training in PH and their beliefs about the future of the PH profession (Supplementary Datasheet 2).

All interviews were conducted over the telephone due to COVID-19 social distancing restrictions and were audiotaped and transcribed verbatim in Hebrew in a standardized format. The interviewer was a Clinical Psychology graduate student, trained in qualitative research methods and supervised by the study's research staff (OB and ZM). The interviewer received the list of 35 PH managers and contacted them to explain the study's purpose and ask if they wanted to participate. Thirty-one managers agreed to participate and were given a detailed explanation of the study plan. No relationship was established between the interviewer and the study's participants before the study commencement. Each interview was audio-recorded and lasted between 40 and 60 mins. The research team discussed the emerging key themes during the interview, and data richness and some of the interviews were analyzed in parallel with the data collection. We derived new hypotheses as a result of this ongoing data analysis.

### Data analysis

**Figure 1:**
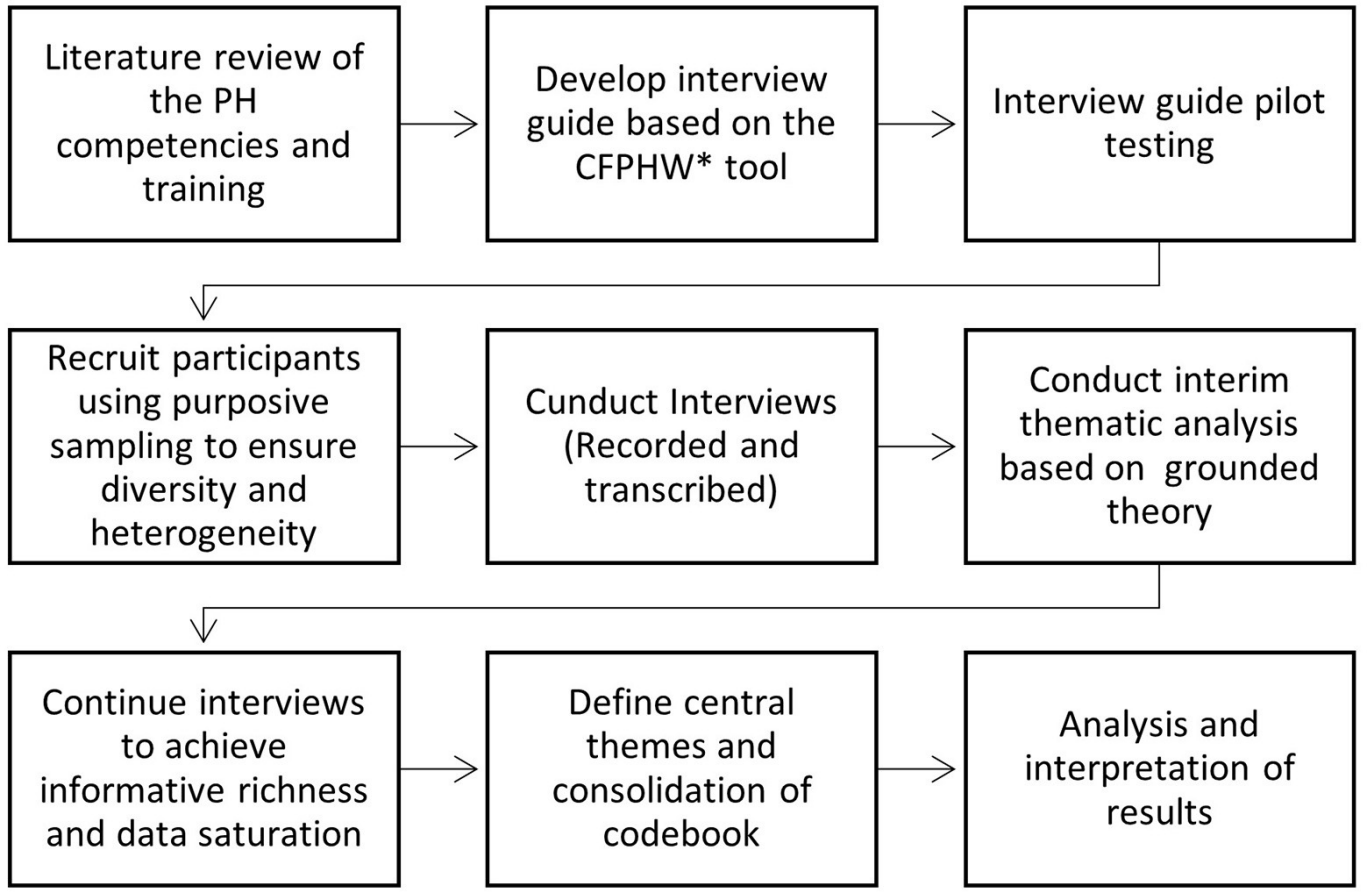
Study procedure from data collection to the synthesis of local analysis. The * symbol indicates the WHO-ASPHER Competency Framework for the Public Health Workforce in the European Region.

All the interviews were transcribed and analyzed by two researchers (OB and ZM) using a thematic analysis method based on grounded theory (16). Grounded Theory is based on concepts that emerge as the theory is formed. The analysis included incorporating deductive themes arising from the research topics and based on an exhaustive literature review of PH competencies and training, together with inductive themes that emerged from the data (17). The transcripts were analyzed in several stages in which the interviews were read at least once to achieve an in-depth and comprehensive knowledge of the data. Next, researchers identified ideas, categories, and themes related to the study's objectives. Subsequently, central themes were redefined to include encoded quotes and examples based on re-reading the transcripts. Relevant passages were marked as a “findspot” and allocated to one of the content themes. We conducted an ongoing internal quality audit, adapted from Mays and Pope (17) and Tong et al. (18), to determine whether the data were collected, analyzed, and reported consistently according to the study protocol. The themes and quotes were translated and documented in English at the final stage. We used a standardized codebook to ensure the validity of the translations from Hebrew to English. Figure 1 illustrates the study procedure.

### Ethics

The interview guide and study were approved by the Ashkelon Academic College Ethics Committee (Approval # 31-2021). Written informed consent was obtained from all participants.

## Results

Twenty-one women and ten men participated in the study. Twenty-two were PH physicians (MD and MPH), four PH nurses (with MPH), three health promotion professionals, and two environmental and food inspectors. Nine interviewees worked at the regional health department, nine at the MoH, three in hospitals, three in research units, five at the headquarters units, one in a health fund, and one in the Israeli Defense Forces Medical Corps.

The data analysis resulted in six main themes that emerged from the interviews: public health workforce's low professional status and the uncertain future of the public health workforce; links between the community and Higher Education institutions; the centricity of communication competencies; need to improve health promotion; the role of leadership, management, and partnership, and innovation in public health. Table 1 illustrates the themes, categories, and quotes related to public health workers.

**Table 1:** Themes, categories, and quotes related to public health workers.

**Themes**	**Category**	**Representative quotes**
Theme I: The Public Health workforce's low professional status and the uncertain future of public health workforce	Workforce crisis	“*The main problem of the profession, I think, is not the training—but the employment afterward. There are too few jobs in the field of public health. Not enough available positions, positions for interns, and experts. It's very hard.” (Interviewee 22, PH Physician)* “*I'm not very optimistic about the future of this whole profession of public health as it is today. It is challenging to recruit staff. If you recruit staff, the worker leaves quickly because other places pay better.” (Interviewee 12, PH Physician)* “*The basis of any organization is its workforce. Currently, in public health, the profession's status is quite low. It reflects the low level of salary, but not only that, but it also reflects the basic format of the profession, which needs a change. We need to redefine the vision. I think we are in the middle of a deep public health workforce crisis.” (Interviewee 19, PH Physician)*
	The need for PH professional growth and development	“*We have to grow; we have to get more power, to be involved in other aspects of health all the time. Not just during an epidemic. We must encourage public health professionals to present their opinions to the public on routine health issues, not just during an epidemic.” (Interviewee 30, Physician, Health Management)* “*Public health is a profession that should be at the forefront. But it suffers from terrible public relations, in my opinion. People do not come to study PH; people do not choose it as a profession. The COVID-19 crisis has trampled on the place of public health. I think it takes time and energy to push it forward. To raise public awareness and self-awareness as well. It is probably also our problem how we perceive ourselves and represent ourselves.” (Interviewee 11, PH Physician)*
Theme II: The links between the community /field and higher education institutions	Gaps between training and practice	“*There are gaps between training and the field. Environmental epidemiology, water, and food interventions, or smoking are issues that are not given enough attention during academic training. Health promotion is very much dependent on what district you work in. There is a large gap.” (Interviewee 19, PH Physician)* “*There is not much training on current issues such as the climate crisis and air quality. This is an issue that is less taught. The training concentrates greatly on basic epidemiology. It would be beneficial if there were further non-academic studies, as there are in all kinds of specializations.” (Interviewee 12, PH and Health Management Physician)*
	Research collaborations	“*We don't have enough skilled workforce in research, epidemiology, and statistics. We have an excellent, incredible database on diseases. We were the first to report the data on the Long Covid in the world. Unfortunately, I did not have people with research competencies.” (Interviewee 25, PH Physician)* “*In training, there are not enough interfaces with other fields in medicine, not enough interfaces and research collaborations with nurses, doctors, and therapists. There is a professional unnecessary separation, and we should change it.” (Interviewee 2, PH Physician)*
	Practical experience	“*Training in public health must be related to an internship or practicum. Implementing practical internships in health departments or medical organizations is necessary. It is not recommended to provide academic training in PH without involving practical experience and research work as part of the training.” (Interviewee 10, Health promotion professional)*
Theme III: The importance of communication competencies	Communicating health messages in different channels	“*The pandemic emphasized the importance of communication training, dealing with politics, and the ability to portray effective messages. All the preoccupation with information and involvement of politics in the field of public health—this is an area that needs more attention.” (Interviewee 19, PH Physician)* “*Decision-makers have ignored us; they think the government is also an expert in epidemiology. We need more communication competencies, knowledge to stand in front of an audience and communicate information to the public using different channels.” (Interviewee 11, PH Physician)*
	Cultural adaptation of messages to the public	“*Communication competencies are competencies that need to be constantly maintained. We should identify communication competencies required in the current period. Everyone is exposed to technology, but there are minority groups who do not use this technology. Training programs should not only identify risk groups but also should learn how to convey health messages to special populations.” (Interviewee 11, PH Physician)*
Theme IV: Improving Health Promotion	Expanding the practice of health promotion	“*I think health promotion needs more training and competencies by using psychological methods, such as motivational tools, coaching, dynamic group facilitation competencies. I mean all these practical tools that help people make the change, help them participate in the processes of change. The Ministry of Health and its employees need to be better trained to work in health promotion. How to talk, how not to scare the public, and how to help people make the change. During the pandemic, they made all possible mistakes along the way and caused more opposition than could have been addressed otherwise.” (Interviewee 17, Health promotion professional)* “*One of the things that need to be done is to plan and evaluate interventions. You teach this [in schools of PH], but you don't always do it in practice. We must focus on planning health promotion interventions and plans adjusted to special populations.” (Interviewee 23, Health promotion professional and PH Physician)*
	Focus on prevention	“*Public health has not been given the proper place. It is time for leaders to understand that more resources should be spent on prevention—it is much more effective than treating diseases. The pandemic did not help us so much because policymakers only dealt with treating patients…., and they forgot the real public health. Health promotion touches all areas and as such should be taught in any course and should be implemented in workplaces and not just taught in academia.” (Interviewee 6, PH Nurse)*
Theme V: Increasing the roles of leadership, management, and partnership	Strengthening partnership	“*Making knowledge accessible to the public and creating a common language, common vision, is very important, but I am talking about the people in the health system, in the education system, in the system of other government ministries. We need partners to carry out all kinds of actions. For example, local authorities. So, if there is a lack of leadership, common language, and common knowledge, that's a problem.” (Interviewee 15, PH Physician)* “*We need to significantly expand the training in various areas and expand the services and partnership in the field of PH and health promotion with the local authorities, strengthen this connection and turn it into something strongly organic. There is no doubt that local authorities have a significant role in PH.” (Interviewee 26, PH and Health Management Physician)*
	Decision-making competencies	“*The COVID-19 crisis showed us all the important need to be well qualified in making decisions under conditions of uncertainty and pressure. To face not-so-simple objections and to know how to discriminate between the essential information and the non-essential.” (Interviewee 21, PH and Health Management Physician)*
	Leadership and management competencies	“*The COVID-19 crisis exposed the deficiencies in competencies such as assertiveness and negotiation, leadership and tackling new national challenges. These competencies need to be strengthened.” (Interviewee 4, PH Nurse)* “*As we saw during the pandemic, it is very important that PH leaders know how to deal with politics and have the ability to carry out effective advocacy. We have also seen this in the past at all sorts of events. During the COVID-19 crisis, we understand how critical it is” (Interviewee 19, PH Physician)*
Theme VI: Innovations in Public Health management	Enhancing innovative computer-based and digital technologies	“*The whole digital issue needs significant improvement. The saddest thing is that Israel is well developed in digital capabilities and technological innovation. Still, we are not sufficiently implementing innovative epidemiological technologies such as computer-based methods.” (Interviewee 4, PH Nurse)* “*I think one of the things we were missing, especially during the COVID-19 crisis, was the issue of information systems. Innovative software that can perform epidemiological calculations is critical, the future will use medical decision support systems, and it must be implemented in public health.” (Interviewee 8, PH Physician)*
	Fostering a strong technological infrastructure for rapid and professional decision-making	“*It is necessary to invest in innovation and create technical and other interfaces to map health status. We should get information about the population's health; that should be the vision. This infrastructure should be extended to other diseases, not only infectious but also cancer, diabetes, and even environmental-related health diseases.” (Interviewee 9, PH Physician)*

### Theme I: Public health workforce's low professional status and the uncertain future of the public health workforce

All interviewees spoke about their concerns related to the perceived low professional status of PHW. They indicated the necessity to invest more resources in training and preserving PHW, highlighting the increased demands for disease prevention, links with excluded communities, and the need for rapid responses to new public health challenges. The decline in the PH budget in recent decades has made it difficult for the PHW to meet routine needs, and these gaps became more significant during the COVID-19 pandemic. The PH system in Israel reached its insufficiency point early during the pandemic, requiring reassigning routine PH operations to other agencies while derailing important ongoing health activities such as health promotion activities or non-COVID-19 vaccinations such as childhood immunization schedules. The cumulative impacts of years of PH system neglect allowed only limited responses to the most urgent ongoing issues while generating workforce burnout, as described by Khouri et al. (19) and many others. Moreover, other health service providers, such as hospitals and health funds, do not train or employ PH professionals and therefore had to rely mainly on the limited PH workforce, which failed to provide the needed responses in a timely fashion. Examples relevant to pre-COVID-19 and during the pandemic included comprehensive epidemiological data analysis, preventive and health promotion work among patients and healthcare workers, including the need to collaborate and build trust with the community and community health workers.

The interviews stressed the importance of improving their employment conditions, increasing salaries, and creating additional job positions, including those suitable for MPH graduates and other allied health professionals who are not medical practitioners. Other interviewees suggested that clinical-oriented medical care organizations (such as hospitals and health funds) should employ many more qualified PH practitioners and engage young doctors and nurses to increase their professional exposure and competency in PH matters.

The interviewees believed that the central roles that PH professionals hold in the MoH could improve the professional status of public health workers in the clinical-oriented medical community. They anticipated that more resources would follow after the post-COVID exposure, given the increased prominence of public health needs during the pandemic.

Several interviewees underscored their general satisfication and devotion to their work and are content with the changes they were able to facilitate in the community while developing professionally. Many interviewees expressed serious concerns about the future of the PH profession. They offered pessimistic views about facing the gaps between the growing needs and the increasing demands for PH and considering the COVID-19 implications. Nevertheless, many highlighted the urgent need to further develop the PH profession's status.

### Theme II: Links between the community/field and higher education institutions

The growing importance of reciprocal partnerships between the “field” and academic higher education institutions was highlighted in several interviews. Interviewees pointed to the gaps between training and practice in the field and raised the urgency to tailor the educational training to the changing community PH needs. Public health research in health departments is currently performed as “self-initiatives” without dedicated resources. In addition, interviewees underscored that there are limited research collaborations among the different healthcare professions.

Applied PH research can be an excellent opportunity to strengthen the trust, respect, and connections between academia and field practitioners while upgrading the PH system, broadening the range of competencies, and enhance professional satisfaction. Lecturers from academia teach in the PH system and establish “in-service training” for PHW staff; however, due to a lack of resources and incentives to encourage research, this mutual “fertilization” is not sufficiently taken advantage of. Interviewees suggested that continuously updating knowledge and strengthening research competencies through lifelong learning and immersive opportunities could improve the professionalism of PHW. Interviewees also recommended exposing PH students to real-world practices in the field during training by visiting organizations that provide PH services. They also suggested that teaching in academic institutions should include problem-based learning and case studies, including how to balance needs and address ethical and resource dilemmas.

The interviewees indicated that PH leadership should be more dynamic and learn to adapt more swiftly to the challenges of the changing world, such as climate change and preparedness for global crises. They recommended that outreach activities with academic institutions be more attentive to the new fields of interest, including acquiring new methodological tools, both quantitative (e.g., integration of big data) and qualitative (including using mixed methods), and train PH students to employ versatile responses by equipping them with the appropriate tools and technologies to address new challenges, such as refugee migration and climate change.

### Theme III: Importance of communication competencies

Communication competencies include informing and influencing individuals, communities, and various audiences across essential health issues (20). The media profession is a distinct domain viewed as far from the remit of PH. However, from the beginning of the COVID-19 outbreak PH professionals were continuously in the media spotlight and PH professionals were expected to possess advanced communication and advocacy competencies using the media channels. All Interviewees discussed the ambiguous attention that PH received from the press. On the one hand, the public and politicians are more aware of PH professionals' central role in health decisions and have become acquainted with basic epidemiologic terms. On the other hand, public frustration regarding the frequent changes in the public policies and management of COVID-19 and criticism about the quality of decisions made at the political level was directed squarely at PH professionals. Interviewees indicated that PH professionals should have robust training in media interviews and learn to communicate scientific and complex ideas to lay audiences in different communities. The importance of PHW competencies that promote resilience to “infodemics” among individuals and communities, including mis/disinformation, and promoting self-efficacy for self-protective health behaviors was reinforced recently by the WHO (21).

Interviewees also noted that PH professionals should better understand how the media works, learn to better respond to crisis events, communicate critical messages, and use practical tools to communicate effectively on different media channels. These channels include social media platforms which had a dominant role during the pandemic, as PH issues were intensely discussed on social media platforms where many misconceptions and conspiracy theories were spread (22).

Interviewees also suggested that PH professionals should portray health messages in a culturally sensitive fashion to diverse populations. It was also mentioned that messages need to reach Israel's social and geographical rural areas.

### Theme IV: Improving health promotion

Participants addressed the need to expand health promotion practice using practical tools for ongoing interventions in the PH field. Several interviewees commented that health promotion and education expertise is not sufficiently used in addressing racism and social inequities. As a result, mistakes were made in communicating the public health-promoting messages related to COVID-19, especially to minorities, migrant workers, and other vulnerable groups.

Interviewees noted that the health system is usually preoccupied with curative treatment rather than prevention efforts. Acute and chronic care overtook the resources and managerial attention needed for health education and promotion or program assessments. Several interviewees commented that more resources were needed to prevent viral transmission and identify health-related risks such as stress, loneliness, obesity, smoking, and chronic diseases. Further, they indicated that COVID-19 management should have employed a more interdisciplinary approach. They also commented that PH professionals should be more active in promoting physical activity, proper nutrition, and stress relief.

### Theme V: Increasing the role of leadership, management, and partnership

Interviewees expressed concerns about the low levels of training in leadership competencies and the ability of managers to build and maintain partnerships and collaborations with other agencies in the community. They emphasized that PH training should include foundation training in management and leadership skills to enable graduates to lead change during times of crisis in the community. PH professionals should improve the links with vulnerable communities and health institutions and partner with local authorities and government agencies to identify health disparities and implement health interventions.

Interviewees mentioned that PH professionals in managerial positions should have improved decision-making competencies in situations of uncertainty. PH training includes evidence-based practice and decisions supported by epidemiological findings. However, during the last two decades and especially during the COVID-19 outbreak, the need for decision-making in states of uncertainty and the need to move to informed decision-making that is shaped and focused on different kinds of evidence became evident. These changes became even more pressing as PH officers had to make swift decisions at the national and local levels based on partial and often conflicting information that became obsolete.

Some interviewees mentioned that it is essential for PH leaders to acquire competencies related to political discourse, collaborate with politicians to prioritize PH issues, and lead changes at the national level with key government stakeholders and politicians.

### Theme VI: Innovations in public health management

Interviewees advised that PH organizations must engender a supportive organizational culture that motivates a competent workforce and supports new technologies to address PH challenges. In particular, this should include using innovative computer-based and digital technologies to monitor and manage recurrent and emerging diseases, chronic morbidity, aging population, societal inequities, unhealthful lifestyles, climate change. They stressed, the skills needed to analyze big datasets of the health funds and hospital discharge information systems of the MoH which are well developed but underutilized in Israel. Interviewees also indicated that the limited technological infrastructure in PH was one of the primary reasons for the deficiencies of the PH system in dealing with the COVID-19 outbreak in Israel. Fostering a robust technological infrastructure is critical for rapid and professional decision-making.

## Discussion

Our findings highlight the lack of investment in the PH workforce and infrastructure which limited the capacity of the PHW in Israel to better respond to COVID-19 due to years of system neglect, budget cuts, and lack of investment in the PH workforce and infrastructure, limiting the capacity of the PHW in Israel to respond to COVID-19 and other emerging health challenges. Our findings illustrate that PH workforce gaps became evident during the COVID-19 crisis and underscored essential competencies required to face new health challenges. Interviewees from research departments, headquarters units, and the MoH pointed to essential competencies that should be taught in PH academic programs, such as communication and health promotion, leadership, and technology-based innovation competencies. At the same time, interviewees from the regional health department and health funds pointed to training competencies that should be strengthened “on the job” with continuous education courses and employer initiatives to keep PH staff updated on current practices, including emerging research and leadership.

Our findings highlight the ongoing weaknesses in the relationships between community-based workers, clinical teams, and university academics regarding shared goals, knowledge, and mutual respect. These “relational dynamics” are associated with a lack of frequent, timely, accurate, and problem-solving communication, predicting low quality and efficiency of their interactions (23). All Interviewees raised the need for improved communication competencies which resonates with Nutbeam (24), who stated that better communication competencies in PH are an essential professional route to increase health literacy, fight health inequalities, and improve the status of marginalized individuals and affected communities. Interviewees emphasized in line with Back et al. (25) the importance of relationships with the media, as was well demonstrated during the COVID-19 pandemic. One of the effective strategies was active participation in daily media and news broadcasts to allay the public's concerns. Media exposure was used as an opportunity to increase the reputation and knowledge of PHW.

Interviewees especially from regional health departments and headquarters units also mentioned that PH professionals should employ an integrated approach to disease prevention. Kickbusch similarly discusses the need for health promotion professionals to understand the nature of pandemics but also the nature of health decision-making around the pandemic, the roles of health organizations and communities in reaction to pandemics, and the significant effects of health promotion in the rapidly evolving management of pandemics (26).

Leadership competencies were a key component for successful management during the COVID-19 outbreak (6). The current study's findings are in line with recent studies that demonstrate the increased demands on PH managers, highlighting the need to train future workers in leadership competencies to meet growing PH challenges (4, 27). Indeed, during the COVID-19 outbreak, PH professionals took on central positions in discussing and framing the practice of science and decision-making and became widely visible to the public. The findings regarding the need for leadership competencies build an other lessons learned from the pandemic as presented by Glenn et al. (28), including recognizing the advantages of the Health in All Policies approach (29), the importance of maintaining a balance between the need for timely evidence-based decision-making in highly uncertain circumstances (30), and the need for a more prominent role for PHW in the public's eye (31). The need for enhanced collaboration and partnerships mentioned by the interviewees was also captured in a Public Health Workforce Interests and Needs Survey conducted by Sellers et al. (32), demonstrating the emerging awareness and importance of science that must be inclusive and equitable, including opportunities for education and capacity development.

PH service leaders need to be actively involved and support research at their institutions and establish fruitful connections with other experts and institutions. Additionally, the concept of shared, evolving knowledge was raised by several interviewees, including those from hospitals and research units, demonstrating the need for the PHW to strengthen the links between practitioners in the field and academic researchers. These links were also mentioned in relation to the importance of PH students' practical experience in PH organizations during studies and have been called out in previous studies (33, 34).

Most of the participants expressed deep concerns about the future of PH. The striking gaps between the primary positions that PH professionals hold in daily-life decisions, including managing COVID-19, and the unattractive working conditions they endure. Low incomes and prestige hinder candidates from considering public health as a career choice. These findings follow a recent examination of the US governmental PHW by Sellers et al. (32), who noted that the salary gap is a significant challenge to recruitment and retention in public health. The lack of a central registry of PH professionals and professional certification detracts from the prestige associated with being a member of the PHW (4, 35, 36).

### Limitations

Our study has several limitations. First, the study results reflect the Israeli health system, which may not be generalized to other countries with their distinct health delivery systems, comprising unique legislative and organizational characteristics, and within different clinical and political settings. Second, although the themes seemed consistent across the interviewees, and the influence of the varying systems on the findings was frequently discussed during the data collection and analysis, the local and specific impacts of these cultural barriers may have been under-appreciated (37). Third, the interviews were transcribed from Hebrew, the native language in Israel. This may have increased the chances for variations in the interpretation of our data. We made all efforts to ensure methodological rigor and validity of the translations from Hebrew to English by using a standardized codebook, meeting frequently, sharing and comparing our results, and performing a pilot analysis. Throughout the study, we conducted an ongoing internal quality audit during our weekly meetings, adapted from Mays and Pope (17) and Tong et al. (18), to determine whether the data were collected, analyzed, and reported consistently according to the study protocol. Finally, interviewees' perceptions may be influenced by participants' personal experiences and working conditions during the COVID-19 pandemic, which seriously challenged the capacity of the PHW in Israel and internationally (19, 38).

### Conclusions and recommendations

We believe that the study considerably advances the understanding and roles of the PHW perceptions often missed in quantitative research and is valuable in presenting a more comprehensive and in-depth picture of the effects of the pandemic on PHW. The critical role of the PHW in responding to and eventually recovering from the COVID-19 pandemic emphasizes the need to strengthen PHW competencies. Capacity building of a well-qualified and competent workforce is crucial to the resilience of the PH system in times of global crisis. Improving preparedness to confront emerging health challenges requires a sustainable PHW, governmental investment in the PH system, improving the profession's prestige and work conditions, and ongoing support for strong public health leadership.

Various steps are required to improve PHW competencies and globally strengthen PH systems. These include establishing a task force composed of PH services professionals and representatives of PH higher education programs to discuss ways to collaborate, redesign and better adapt educational programs to the field's needs. PH training programs should include new learning skills, encouraging innovative thinking, self-learning skills, problem-based learning (39), and case simulations to enhance opportunities for advanced education and capacity development. A special emphasis should be put on PH competencies related to outreach to vulnerable sectors of the population at risk that needs health promotion support. In addition, the PH services jointly with the national Higher Education Committees should develop a standardized accreditation process to evaluate PH competencies needed and taught in PH higher education training programs. A robust and competent PHW with extended responsibilities and prominent leadership is necessary to promote population health, including in underserved communities. This will enable the health system to respond more effectively and efficiently to future disasters and emergencies.

## Data availability statement

The original contributions presented in the study are included in the article/Supplementary material, further inquiries can be directed to the corresponding author/s.

## Ethics statement

The studies involving human participants were reviewed and approved by Ashkelon Academic College Ethics Committee (Approval # 31-2021). The patients/participants provided their written informed consent to participate in this study.

## Author contributions

OB, RO, LL, KC, KD, TT, and ZM: conceptualization. OB, RO, LL, KC, and ZM: project administration. OB, KD, and ZM: data curation. OB and ZM: analysis, interpretation, and writing-original draft. OB, RO, LL, KC, PB, ND, KD, MD, LO, FM, YN, MR, TT, and ZM: review and editing. All authors contributed to the article and approved the submitted version.

## Funding

The current study is part of a multinational Erasmus+ Capacity Building in Higher Education funded project- Sharing European Educational Experience in Public Health for Israel (SEEEPHI): harmonization, employability, leadership, and outreach. The project is financed by EU funds within the framework of the Erasmus+ Programme of the European Union (Grant Agreement 618578-EPP-1-2020-1-BE-EPPKA2-CBHE-JP).

## Conflict of interest

The authors declare that the research was conducted in the absence of any commercial or financial relationships that could be construed as a potential conflict of interest.

## Publisher's note

All claims expressed in this article are solely those of the authors and do not necessarily represent those of their affiliated organizations, or those of the publisher, the editors and the reviewers. Any product that may be evaluated in this article, or claim that may be made by its manufacturer, is not guaranteed or endorsed by the publisher.
