# Supplementary material for: Emerging lessons from the COVID-19 pandemic about the decisive competencies needed for the public health workforce: A qualitative study
Source: Front Public Health. 2022 Sep 2;10:990353. doi: 10.3389/fpubh.2022.990353 (PMC9479633; doi:10.3389/fpubh.2022.990353)
Supplement: Supplementary file 1 [file Data_Sheet_1.PDF]

Supplementary 1 – Interviewee's characteristics

Participant	Organization	District	Role
1	Regional health department	North periphery	PH Physician
2	(Ministry of Health (MOH)	Center	PH Physician
3	Hospital	North periphery	PH Physician
4	Research unit	Center	PH Nurse
5	MoH	Center	PH Nurse
6	Regional health department	South Periphery	PH Nurse
7	Headquarters unit	Center	PH Nurse
8	Regional health department	North periphery	PH Physician
9	Regional health department	Center	PH Physician
10	Hospital	Center	Health Promotion professional
11	Israel Center for Disease Control (ICDC), MoH	Center	PH Physician
12	Regional health department	Center	PH and Health Management Physician
13	MoH	Center	Environmental inspector
14	MoH	Center	PH Physician
15	Regional health department	North periphery	PH Physician
16	Research Institute	Center	PH Physician
17	Health Fund	Center	Health Promotion professional
18	Headquarters unit, University	North periphery	Health Promotion professional
19	Regional health department	Center	PH Physician
20	Headquarters unit	Center	PH Physician
21	Regional health department	North periphery	PH and Health Management Physician
22	Research Institute	Center	PH Physician

23	Headquarters unit	Center	PH Physician, Health Promotion professional
24	Israel Center for Disease Control (ICDC), MoH	Center	PH Physician
25	Israeli Force Defense	Center	PH Physician
26	MoH	Center	PH and Health Management Physician
27	MoH	Center	PH Physician
28	MoH	Center	PH Physician
29	Regional health department	Center	Environmental and food inspector
30	Hospital	South Periphery	Physician, Health Management
31	Headquarters unit	Center	PH Physician
