# Supplementary material for: Emerging lessons from the COVID-19 pandemic about the decisive competencies needed for the public health workforce: A qualitative study
Source: Front Public Health. 2022 Sep 2;10:990353. doi: 10.3389/fpubh.2022.990353 (PMC9479633; doi:10.3389/fpubh.2022.990353)
Supplement: Supplementary file 2 [file Data_Sheet_2.PDF]

Supplementary 2 – Study Interview Guide

1. Tell me a little about yourself, what is your profession, where do you work, how many years have you been in your current position? How many years have you been in management positions in the health system / in the PH services?
2. I want to ask you if you feel there is a lack of adequate training for public health workers on issues such as (give examples from the CFPHW survey):
    a. Science & Practice
    b. Promoting health
    c. Law, Policy & Ethics
    d. One Health & Health Security
    e. Leadership & Systems Thinking
    f. Collaboration & Partnerships
    g. Communication, Culture & Advocacy
    h. Governance & Resource Management
    i. Professional Development & Reflective Ethical Practice
    j. Organizational Literacy & Adaptability
3. Are there any additional points /issues in which you think there isn't currently adequate training for public health workers?
4. Do you think that the COVID-19 crisis has revealed the need for new competencies and competencies required of public health workers?
5. How do you see the PH services in the future? What do you think we should invest more in? What barriers exist today to the development of the PHW?
